# Applying High-Speed Vision Sensing to an Industrial Robot for High-Performance Position Regulation under Uncertainties

**DOI:** 10.3390/s16081195

**Published:** 2016-07-29

**Authors:** Shouren Huang, Niklas Bergström, Yuji Yamakawa, Taku Senoo, Masatoshi Ishikawa

**Affiliations:** Graduate School of Information Science and Technology, University of Tokyo, 7-3-1 Hongo, Bunkyo-ku, Tokyo 113-8656, Japan; niklas_bergstrom@ipc.i.u-tokyo.ac.jp (N.B.); Yuji_Yamakawa@ipc.i.u-tokyo.ac.jp (Y.Y.); Taku_Seno@ipc.i.u-tokyo.ac.jp (T.S.); Masatoshi_Ishikawa@ipc.i.u-tokyo.ac.jp (M.I.)

**Keywords:** high-speed vision sensing, fast and accurate robotic position regulation, industrial robots, dynamic compensation, add-on module, compensation of uncertainties

## Abstract

It is traditionally difficult to implement fast and accurate position regulation on an industrial robot in the presence of uncertainties. The uncertain factors can be attributed either to the industrial robot itself (e.g., a mismatch of dynamics, mechanical defects such as backlash, etc.) or to the external environment (e.g., calibration errors, misalignment or perturbations of a workpiece, etc.). This paper proposes a systematic approach to implement high-performance position regulation under uncertainties on a general industrial robot (referred to as the main robot) with minimal or no manual teaching. The method is based on a coarse-to-fine strategy that involves configuring an add-on module for the main robot’s end effector. The add-on module consists of a 1000 Hz vision sensor and a high-speed actuator to compensate for accumulated uncertainties. The main robot only focuses on fast and coarse motion, with its trajectories automatically planned by image information from a static low-cost camera. Fast and accurate peg-and-hole alignment in one dimension was implemented as an application scenario by using a commercial parallel-link robot and an add-on compensation module with one degree of freedom (DoF). Experimental results yielded an almost 100% success rate for fast peg-in-hole manipulation (with regulation accuracy at about 0.1 mm) when the workpiece was randomly placed.

## 1. Introduction

Position regulation with respect to objects of interest in terms of set-point positioning or trajectory tracking is a fundamental function of an industrial robot. For robots deployed in the manufacturing industry, position regulation at a fast speed and satisfactory accuracy leads to high productivity, and the ability to adapt to many uncertainties leads to task flexibility and efficiency that eventually helps reduce cost.

Traditionally, there are two main ways of commanding (programming) an industrial robot to implement position regulation: off-line teaching playback, and on-line sensor-guided methods. Off-line teaching playback using a teaching pendant, or physically positioning a robot with a teaching arm, is the more widely used method. The method features a user-friendly interface developed by commercial robot manufacturers, and is applicable to various tasks that require no knowledge on the part of the user of the mapping between the user-concerned task space and the actuation-concerned joint space. It is usually motion optimized and reliable so long as task conditions do not change. As detailed in [1], negative effects of nonlinear dynamics during high-speed motion may be pre-compensated in order to achieve accurate path tracking during the playback phase. However, it is impossible for a playback robot to adapt to significant variations in the initial pose of a working target or unexpected fluctuations in the pose of the target during manipulation by relying only on internal sensors, such as encoders [2]. Although it is easy to implement CAD model-based teaching, it is rather time consuming and sub-optimal if a large number of teaching points requiring high accuracy are to be manually taught. In [2], a view-based teaching playback method was proposed to achieve robust manipulation against changes in task conditions. In this method, a mapping from recorded images to recorded movements in the teaching phase is rendered by an artificial neural network (ANN). In the playback phase, the motion of the robot is determined by the output of the neural network calculated through scene images. However, the approach is difficult for teaching jerky robot motion and cannot be applied to cases where high motion accuracy is required.

On the contrary, by using real-time sensor-guided (e.g., vision, force sensor, etc.) feedback control, the teaching task can be eliminated and the robot can adapt quickly to uncertainty in the working environment. Accurate sensor-motor models are typically used to implement position regulation. Many adaptive approaches have also been proposed (e.g., [3,4,5,6,7,8]) to address the control problem in the presence of uncertainty associated with the robot’s mechanical dynamics, or with sensor-robot mapping. However, a common disadvantage of on-line sensor-guided robots is that, due to their mechanical inertia as well as nonlinear dynamics, they react relatively slowly to changes in target information captured by the sensor system [9]. It is usually difficult to obtain satisfactory accuracy at a high speed due to the complex dynamics and large mechanical inertia of an industrial robot. There are other implementation issues with the on-line sensor-guided approach. Since a commercial robot’s controller is packaged as a “black-box”, it is usually impractical for users to directly access its internal control loops to compensate for nonlinear dynamics. Moreover, the matching between the feedback frequency of external sensors and the control frequency of the robot’s controller is an issue, especially when the robot’s control frequency is not as fast as the feedback frequency of external sensors.

**Figure 1:**
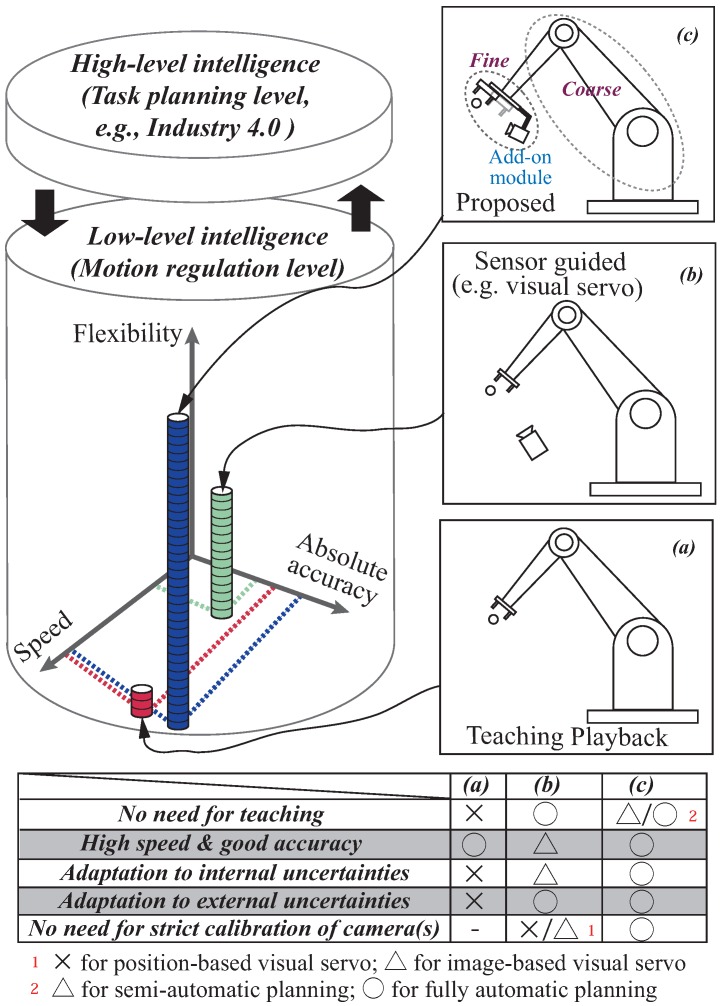
Summary of major programming methods for, and the hierarchical intelligence structure of, an industrial robot. (**a**) Traditional teaching playback method, which is poor for task flexibility; (**b**) On-line sensor-guided method, which has better task flexibility but lower regulation speed; (**c**) Proposed method with a coarse-to-fine strategy. By cooperating with the industrial robot using an add-on module that utilizes high-speed vision sensing, satisfactory performance in terms of task flexibility can be obtained while simultaneously achieving fast and accurate position regulation.

To this end, there is a need for a better and more practical solution to the problem of instructing an industrial robot to implement fast and accurate position regulation with sufficient flexibility. From the perspective of compatibility with prevalent commercial robots used in manufacturing, a combination of the advantages of both the teaching playback and the on-line sensor-guided methods is beneficial. Easy integration with a commercial robot’s black-box controller is also an important issue that we consider in this study. Based on the dynamic compensation approach [10], we propose a systematic approach for high-performance position regulation under uncertainty. The method targets a general industrial robot with minimum manual teaching effort, and is based on a coarse-to-fine strategy by introducing an add-on module. The add-on module consists of a 1000 Hz vision sensor and a high-speed actuator to compensate for the accumulated uncertainties while the main robot only focuses on fast but coarse motion. For a greater understanding of the overall objective of this study, a comparison between traditional methods and our proposed method is summarized in Figure 1. The proposed method (Figure 1c) can implement high-performance position regulation while exhibiting satisfactory task flexibility. Flexibility here means the ability to adapt to uncertainties due to the external environment (e.g., calibration errors, misalignment of workpieces, etc.) and the industrial robot (e.g., a mismatch of dynamics, mechanical defects such as backlash, etc.), as well as requiring less calibration work for cameras and a minimum teaching effort. Flexibility is as an important aspect of what we call the “low-level intelligence” of the hierarchical intelligence structure for industrial robots, and we consider it the foundation needed to implement “high-level intelligence” scenarios, such as the *Industry 4.0* [11].

The proposed method is implemented based on a coarse-to-fine strategy inherited from the macro–micro method [12,13]. The macro–micro concept was proposed several decades ago with the aim of enhancing system bandwidth for rigid manipulators and suppressing bending vibrations for flexible manipulators. It should be noted that although similar coarse-to-fine strategies based on the macro–micro method have been proposed in related research (e.g., [14,15,16,17]), this work differs from them mainly in the following two aspects: first, the proposed method provides a cost-effective solution in terms of system integration and easy implementation, which enables low-cost robots to realize high productivity. Second, by introducing high-speed vision sensing, action-level intelligence in terms of adapting to systems and external uncertainties is conveniently realized in order to achieve satisfactory flexibility and efficiency.

The rest of the paper is organized as follows: the proposed method with an intuitive analysis of compensating for uncertainties, as well as motion planning for the main robot, is addressed in Section 2. An application implementation concerning a dynamic peg-and-hole alignment is presented in Section 3. Experiments were conducted using a commercial parallel-link robot and an add-on module prototype with one degree of freedom (DoF). The conclusions of this study are presented in Section 4.

## 2. Proposed Method

In this section, we provide an intuitive analysis of the theoretical basis for the proposed dynamic compensation method using high-speed vision sensing, and describe the motion planning method for coarse position regulation in the main robot.

### 2.1. Intuitive Analysis of Dynamic Compensation Method

The proposed dynamic compensation method is based on fusing high-speed visual sensing with a high-speed compensation mechanism. In order to grasp the basic idea, an intuitive analysis is provided with the assumption that the entire system is regulated in image space.

**Figure 2:**
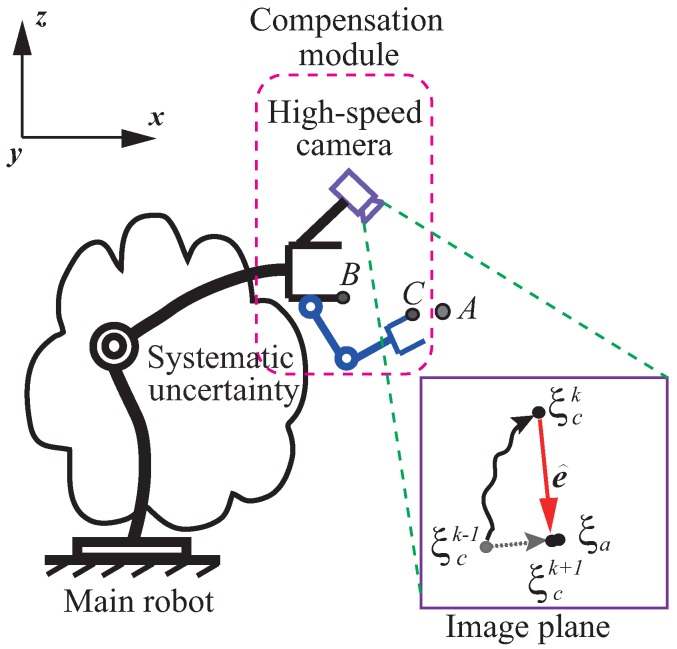
The concept of dynamic compensation. Tool point *C* is controlled to align with target position *A* with the help of a compensation module, although the position of *B* is not certain due to systematic uncertainty in the main robot.

As shown in Figure 2, an arbitrary commercial robot is controlled toward set-point position *A* (for the ease of understanding, the target is assumed to be motionless) at high speed. A direct-driven compensation module is configured to its end effector. Initially, tool point *B* is assumed to overlap with tool point *C*. We refer to image features of target *A* and those of the robot’s tool as $\xi_{a}$ and $\xi_{c}$, respectively, from visual feedback, and error ***e*** for regulation is noted as

(1) $$e=\xi_{c}-\xi_{a}$$

Noting that ${\dot{\xi }}_{c}$ can be divided into two parts, motion effects corresponding to the main robot and the compensation module,

(2) $$\begin{array}{cc} {\dot{\xi }}_{c} & =J_{r}{\dot{\theta }}_{m}+J_{c}{\dot{\theta }}_{c} \end{array}$$

where ${\dot{\theta }}_{m}$, ${\dot{\theta }}_{c}$ represent joint velocity vectors of the main robot and the compensation module, respectively, and $J_{r}$ and $J_{c}$ are the Jacobians (mapping from joint space to image space) of the main robot and the compensation module, respectively. We assume that the compensation module is not activated (${\dot{\theta }}_{c}=0$), and stays still to retain the overlap between *B* and *C*. In the ideal case, the exponential convergence of error regulation (for instance, $\xi_{c}^{k-1}$ converges to $\xi_{a}$ in Figure 2) can be obtained if we apply feedback control, such as

(3) $$\begin{array}{c} {\dot{\theta }}_{m}=-\omega J_{r}^{+}e \end{array}$$

where *ω* is a constant positive-definite coefficient and $J_{r}^{+}$ represents the pseudo-inverse of $J_{r}$. However, in practice, the ideal visual-motor model of $J_{r}^{+}$ for an industrial robot is not available, and is usually estimated with errors due to systematic uncertainties and inaccurate camera calibration. We denote the uncertain part as $\Delta J_{r}^{+}$. The error dynamics with feedback control then become:

(4) $$\begin{array}{c} \begin{array}{cc} {\dot{\xi }}_{c} & =-\omega J_{r}\left(J_{r}^{+}+\Delta J_{r}^{+}\right)e\\ \dot{e} & =-\omega e-\delta \end{array} \end{array}$$

where δ represents the projected uncertainty in image space. In this case, for instance, $\xi_{c}^{k-1}$ moves to $\xi_{c}^{k}$ rather than $\xi_{a}$ in Figure 2 due to uncertainty. It should be pointed out that in spite of the uncertain term δ, the system is still assumed to conduct coarse positioning in the direction of the neighborhood of the target.

Now, let the compensation module be activated with motion ${\dot{\theta }}_{c}$, and suppose $J_{c}{\dot{\theta }}_{c}=\hat{\delta }$, with $\hat{\delta }$ representing the estimation of δ. The closed-loop system then becomes:

(5) $$\begin{array}{c} \begin{array}{cc} \dot{e} & =-\omega e+\hat{\delta }-\delta \\ & =-\omega e-\tilde{\delta } \end{array} \end{array}$$

where $\tilde{\delta }=\delta -\hat{\delta }$. In order to obtain the update law for $\hat{\delta }$, we choose the following Lyapunov function candidate:

(6) $$\begin{array}{cc} V(e,\delta ) & =e^{T}Pe+{\tilde{\delta }}^{T}\Gamma^{-1}\tilde{\delta } \end{array}$$

where *P* and Γ are two symmetric positive-definite matrices. Notice that the direct-driven compensation module is feedback controlled by 1000 Hz of high-speed vision, and the uncertain term δ can be approximated as a constant unknown term during the 1 ms feedback control cycle. Therefore, $\dot{\tilde{\delta }}=-\dot{\hat{\delta }}$, and the time derivative of *V* is given by

(7) $$\begin{array}{c} \begin{array}{cc} \dot{V} & ={\dot{e}}^{T}Pe+e^{T}P\dot{e}-2{\tilde{\delta }}^{T}\Gamma^{-1}\dot{\hat{\delta }}\\ & =-2\omega e^{T}Pe-2{\tilde{\delta }}^{T}\Gamma^{-1}\left(\Gamma Pe+\dot{\hat{\delta }}\right) \end{array} \end{array}$$

Apparently, $\dot{V}=-2\omega e^{T}Pe\leq 0$ if we choose the update law for $\hat{\delta }$ as

(8) $$\begin{array}{c} \dot{\hat{\delta }}=-\Gamma Pe \end{array}$$

From the analysis above, we claim that asymptotic convergence is achievable using the proposed dynamic compensation in spite of systematic uncertainty in the main robot. Several issues should be noted here. First, the same conclusion can be drawn no matter how the main robot is controlled (in task space, as here, or joint space), and compensation capability can be further enhanced due to the fact that the control frequency of most commercial industrial robots is smaller than the 1000 Hz feedback control of the directly driven compensation module. Second, although dynamics are not fully incorporated within the analysis, our claim is still reasonable under the condition that the compensation actuator has a different bandwidth from that of the main robot. Third, although we have assumed the target to be motionless above, it is reasonable to apply the same analysis to cases where the target is moving but its motion is negligible in the context of 1000 Hz high-speed vision sensing. Moreover, although several robust and adaptive control approaches have been proposed (e.g., [3]) for direct control of robots with uncertain kinematics and dynamics, we note the advantages of our method in following two aspects:

1. The method here decouples the direct-driven compensation module and the main industrial robot, and requires no changes to the main robot’s controller. On the contrary, traditional adaptive control methods need to directly assess the inner loop of a robot’s controller (mostly not open), which is usually considered difficult both technically and practically.
2. It is difficult for traditional adaptive control methods to realize high-speed and accurate adaptive regulation due to the main robot’s large inertia and complex nonlinear dynamics. With the philosophy of motion decoupling as well as adopting high-speed vision to sense the accumulated uncertainties, the proposed method here enables a poor-accuracy industrial robot to realize high-speed and accurate position regulation by incorporating a ready-to-use add-on module.

To summarize, the proposed dynamic compensation involves three important features:

1. The compensation module should be controlled accurately and sufficiently fast. Ideally, it has a much larger bandwidth than that of the main robot.
2. The visual feedback should be high speed in order to satisfy the assumption $\dot{\tilde{\delta }}=-\dot{\hat{\delta }}$.
3. The error value ***e*** is the relative information between the robot’s tool point and the target in image coordinates, which can be observed directly.

Finally, it should be noted that since the add-on module works independently of the main robot’s controller, optimal control of the system is difficult to address, and is beyond the scope of this study.

### 2.2. Motion Planning for the Main Robot’s Coarse Position Regulation

The motion of the main robot can be either planned in a semi-automatic manner by coarse and easy teaching, or in a fully automatic way by utilizing visual information. They share the same objective of realizing high-speed motion that is coarse but robust while demanding minimum technical knowledge and workload to program an industrial robot.

**Figure 3:**
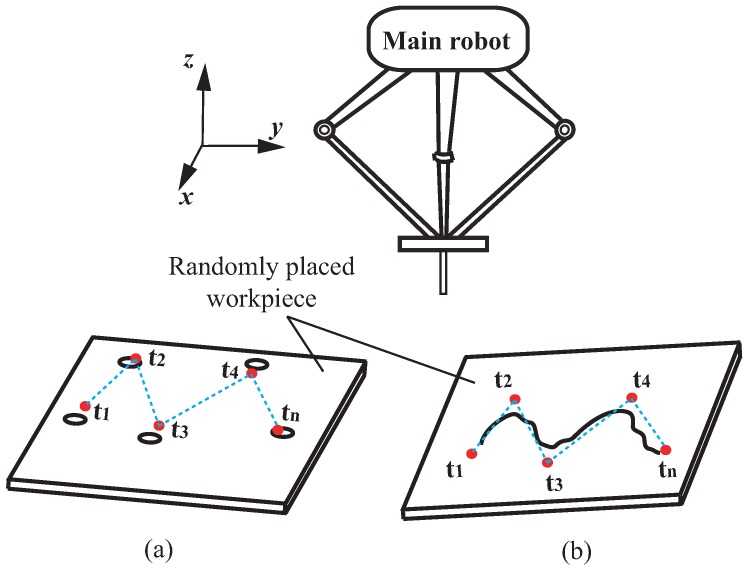
Motion planning for the main robot’s coarse position regulation. Task example: (**a**) Peg-and-hole alignment; (**b**) Contour (path) tracking. For either tasks, semi-automatic and fully automatic methods can be easily implemented. In the semi-automatic method, we simply teach $t_{1}\;...\;t_{n}$ roughly according to the work range of the add-on module (compensation is performed along the *x*-direction). In the fully automatic method, key points $t_{1}\;...\;t_{n}$ are extracted from image coordinate by detecting holes’ center positions in task (**a**) or by line detection and segments approximation in task (**b**), and then they are transferred into the robot’s coordinates.

In the semi-automatic method, key points (via-points) are taught coarsely and randomly. For task of peg-and-hole alignment, it is straightforward as we simply teach each hole’s rough position $t_{1}\to t_{2}\;...\to t_{n}$ as shown in Figure 3a. For other tasks such as tracking a complex unknown paths, as shown in Figure 3b, the main robot is programmed through off-line teaching with a coarse path $t_{1}\to t_{2}\;...\to t_{n}$ along the target contour. Note that random teaching points are picked under the assumption that target holes or target paths are always kept within the work range of the compensation module. As a consequence, the teaching points can be sparse, and off-line programming can be very simple. Uncertainties due to the main robot as well as the environment can be compensated for by the add-on module. The implementations and results for path tracking tasks can be found in our related study [18].

In the fully automatic approach, the main robot’s motion is planned using visual feedback information prior to task execution. Unlike traditional on-line visual servo methods that aim to realize accurate position regulation toward a target (point or path) based on accurate models of image-motor mapping and system dynamics, we only aim for coarse position regulation toward the target. The implementation involves two aspects: a rough calibration for the mapping between the coordinates of the image and those of the robot, and image processing to extract key points (such as hole center or via-points for a path) according to the specified task. An example of the implementation of this method, involving a peg-and-hole alignment task, is described in Section 3.2.

## 3. Application Scenario: Dynamic Peg-and-Hole Alignment

### 3.1. Task Illustration and Experimental System

The dynamic peg-and-hole alignment task was conducted to test the efficiency of the proposed method in realizing fast and accurate position regulation under internal and external uncertainties.

**Figure 4:**
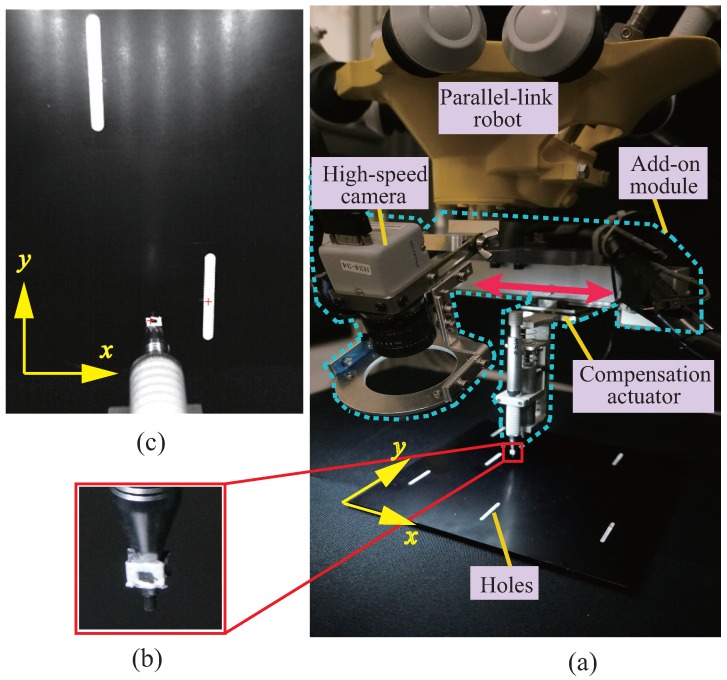
Experimental system. (**a**) Overall setup. A one-DoF (*x*-direction) add-on module and a commercial parallel-link robot; (**b**) Marker representing the peg’s position; and (**c**) Detected marker and reference position (center of the nearest hole).

The experimental testbed is shown in Figure 4. The add-on compensation module had one DoF. A workpiece (metal plate with six randomly configured holes) was blindly placed on a desk for each experiment trial. The holes were 2 mm along the *x*-direction, and were elongated in the *y*-direction to account for the fact that compensation was carried out only in the *x*-direction. A mechanical pencil with a diameter of 1.0 mm acting as the peg was attached to the linear compensation actuator, and the insertion action was driven by an on-off solenoid. The insertion action was activated only if the error between the peg and the center of the hole in the *x*-direction was smaller than 0.8 pixels (corresponding to 0.112 mm) and lasted for more than 0.02 s. The insertion lasted for 0.3 s.

We sought to insert the peg at the center of these holes. As can be seen from Figure 4, the holes formed the white parts of the otherwise black workpiece. Section 3.2 describes the process of detecting and obtaining the positions of these holes.

#### 3.1.1. Robot Systems

**Figure 5:**
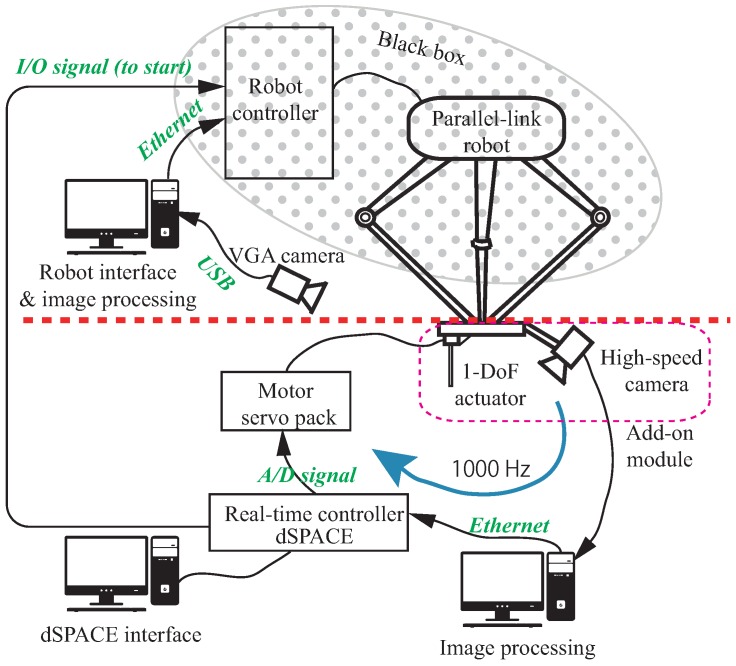
Control scheme. The control system of add-on module operating at a frequency of 1000 Hz was independent of that of the main robot. The main robot’s control system was interpreted as the black box, which renders the proposed method applicable to an arbitrary industrial robot.

A four-DoF parallel-link robot capable of high-speed motion was deployed as the main robot to execute the coarse-but-high-speed motion. As shown in Figure 5, the control system of the add-on module was independent of that of the parallel-link robot, and the main robot could have been replaced with any other industrial robot without changing any other part. A one-DoF linear compensation actuator was used as the add-on module with specifications according to Table 1. In accordance with the proposed dynamic compensation concept, the actuator was designed with large acceleration capability as well as being lightweight. Note that the entire stroke was not used in the experiment, as an eye-in-hand high-speed camera was configured with a field of view of approximately 70 mm within the motion range of the compensation actuator. Therefore, we had an approximate conversion of 1 pixel as 0.14 mm.

#### 3.1.2. High-Speed Vision Sensing

A Photron IDP-Express R2000 high-speed camera [19] (made by PHOTRON, Tokyo, Japan) was used with the eye-in-hand configuration. The camera is capable of acquiring 8-bit monochrome or 24-bit color frames with the resolution 512×512 pixels at a frame rate of 2000 fps. It was connected to an image processing PC (OS: Windows 7 Professional, CPU: 24 core, 2.3 GHz Intel Xeon, Memory: 32 GB, GPU: NVIDIA Quadro K5200) (made by DELL Inc., Round Rock, TX, USA). For the purpose of the experiments in the paper, we set the frame rate to 1000 fps.

Since control was limited to one dimension (along the *x*-direction in our case), the peg and the holes only needed to be aligned along the *x*-axis in the images. The peg was tracked using a marker fixed on the mechanical pencil at some distance from its tip (See Figure 4b). In the captured images, it corresponded to roughly 9×9 pixel patches, and we employed a simple template-based search to find the location of the marker by minimizing the mean squared error. Following marker identification, we found the location at sub-pixel accuracy by computing image moments on the center patch. After locating the peg, we searched for the hole on a row in the image at a fixed distance from the detected marker in the *y*-direction. This image row was effectively binarized apart from the edge regions around the holes. We therefore searched for consecutive white regions (holes) and selected the one with center closest to the peg in the *x*-direction. The hole position was also computed with sub-pixel accuracy using image moments over the non-black region on the searched row. The processing ran within a millisecond using CUDA [20] to enable 1000 fps tracking of the positions of the pen and the hole.

The high-speed camera is configured in such a away that the peg and each hole are both visible, and the relative error in image coordinates is sent to the real-time controller (see Figure 5) by Ethernet at a frequency of 1000 Hz. Since the high-speed camera is configured as the eye-in-hand, uncertainties due to the main robot as well as the external environment can be resultantly perceived as the variations of the hole’s position and they are accumulated within the relative error towards the peg. This is consistent with the dynamic compensation method addressed in Section 2. Note here that the uncertainty of the main robot has mainly resulted from the backlash of the rotational joint, which can be seen from the convergence profile of the main robot’s position regulation addressed in Section 3.4.2.

### 3.2. Fully Automatic Motion Planning of the Main Robot

In the proposed method, an additional low-cost VGA (Video Graphics Array) camera (made by SONY, Japan) was mounted on the frame of the system and directed at the workspace. Using this camera, we fully automate the teaching task by detecting the rough position of holes in the main robot’s coordinates. For this, which, in turn, enabled motion generation in the main robot, two things were required: the relationship between the VGA camera image and the main robot’s workspace needed to be roughly calibrated, and the positions of the holes needed to be detected from the image, and transferred to the main robot’s coordinates using the calibrated relationship.

#### 3.2.1. Simple Calibration Procedure

The first thing to notice was that due to the proposed method, an exact calibration was unnecessary. As such, we did not worry about the intrinsic calibration of the VGA camera. Calibration simply involved computing the planar homography between the workpiece (a metal plate) in the main robot’s coordinates and the image plane of the VGA camera. This was done by letting the main robot move to four points $X_{i}={\left\{x_{i},y_{i},k_{i}\right\}}^{T}$ directly above the workpiece and marking the corresponding locations $X_{i}^{\prime }={\left\{x_{i}^{\prime },y_{i}^{\prime },k_{i}^{\prime }\right\}}^{T}$ in the image. Here, the points $X_{i}$ and $X_{i}^{\prime }$ are homogeneous coordinates representing the 2D coordinates ${\left\{x_{i}/k_{i},y_{i}/k_{i}\right\}}^{T}$ and ${\left\{x_{i}^{\prime }/k_{i}^{\prime },y_{i}^{\prime }/k_{i}^{\prime }\right\}}^{T}$. The four points could be chosen randomly such that no three points were collinear. Using these four point correspondences $X_{i}↔X_{i}^{\prime }$, we computed the homography $H\in {ℜ}^{3\times 3}$ [21] by

(9) $$\begin{array}{c} X_{i}^{\prime }=H\cdot X_{i}\left(i=1,...,4\right) \end{array}$$

Since we only needed a coarse homography, the procedure of choosing four point correspondences was rough and easy to implement. While we, in this paper, only consider one-dimensional compensation, the same calibration procedure applies to full 3D compensation over a limited depth range so long as holes on the workpiece are observable by the high-speed camera for fine compensation. Hence, it would not be necessary to obtain 3D measurements using a stereo camera configuration, or some similar mechanism.

Since the camera was fixed in relation to the main robot’s coordinate frame, the calibration procedure was implemented once, and only needed to be performed again if the camera was moved, or if the height of the workspace changed drastically.

#### 3.2.2. Hole Detection and Trajectory Planning

Usually, the model of holes on the workpiece should be known in order to detect them and calculate their locations. Here, we simplified the detection problem by utilizing the fact that the holes formed the white area of the black workpiece. The holes were identified and their locations were computed using image moments. The resulting points were transferred to the main robot’s coordinate system using the homography computed in Section 3.2.1. Since the calibration mapping between the image and the robot’s coordinates was rough, the detected points expressed in the robot’s coordinates should reside in the neighbor area of their corresponding hole. Following this, the shortest path connecting all of these points was calculated as a traveling salesman problem (TSP) [22]. Finally, the route (all points in order) was sent to the controller of the main robot to generate the corresponding motion, with the motion speed set at 2000 mm/s.

It is worth noting that the path of the motion of the main robot should be planned according to the task at hand. For instance, one can imagine other kinds of tasks, e.g., a welding task, where the robot needs to continuously move according to some desired path. In this case, it would be sufficient to find a few points along the path and let the main robot traverse them in a smooth trajectory. Even if this trajectory does not coincide with the actual path, the compensation will take care of the errors. The computation of such a trajectory is, however, beyond the scope of this paper, and is left for future work.

### 3.3. Fine Compensation of the Add-on Module

Using the update law of $\hat{\delta }$ by Equation (8), the control law of the compensation module may be further obtained theoretically, and the issue of the dynamical impact of the main robot on the compensation module is negligible under the assumption that the compensation module ideally has a larger bandwidth than the main robot. However, in reality, this assumption is difficult to satisfy, and the issue of dynamical impact should still be taken into account. Otherwise, it can undermine the foundation of the coarse-to-fine strategy. In order to realize fine compensation in the context of the proposed coarse-to-fine strategy, a pre-compensation fuzzy logic control (PFLC) algorithm has been proposed in our previous study [23]. The PFLC method is model-independent and includes two cascade fuzzy inference systems (FIS), one for pre-compensation and the other for error regulation. The first FIS was actually to combine the velocity information of the main robot and the compensation module by utilizing the high-speed vision sensing. Its output was then taken as an input to the second FIS to generate regulation force needed for driving. With this method, partial counteraction of the disturbance from the main robot was realized without knowing the explicit mathematical models of the system, and the effectiveness has been verified by simulation and experimental evaluations. Note that the fine compensation of the add-on module was not activated until the target entered the work range of the compensation module, which was guided by the main robot’s coarse and fast positioning. More details of the PFLC algorithm, including implementation and a comparison between PFLC and other non-model-based approaches, can be found in [23].

### 3.4. Experimental Results

#### 3.4.1. Evaluation of Coarse Motion Planning

In order to check the accuracy of the main robot’s coarse motion planning based on the fully automatic planning detailed in Section 3.2, two evaluations were conducted. First, several rounds of rough calibration were carried out with the same workpiece pose to check deviations due to varying calibrations. Second, the workpiece was placed randomly in different positions to check for error distribution with respect to space under the same calibration. Note that the error due to image recognition for the position of each hole was sufficiently small to be negligible in these evaluations.

**Figure 6:**
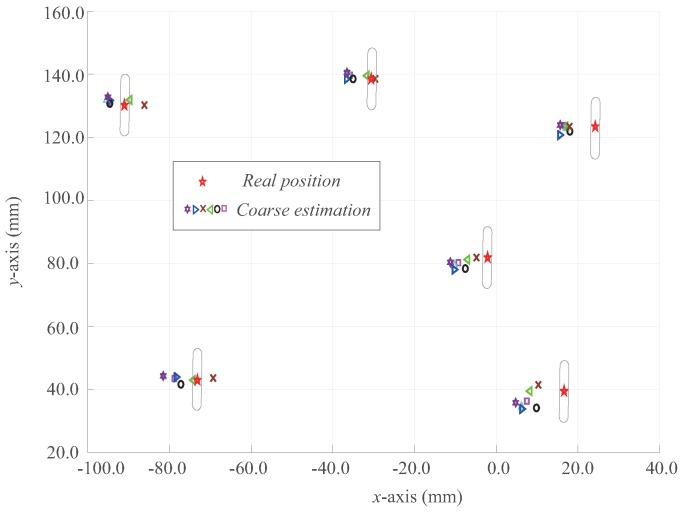
Deviations in the positions of the holes due to varying calibration.

The result of the first evaluation are shown in Figure 6. We see that, with different calibrations, the deviations in the centers of the holes were within a certain range, although the calibration points for each round were picked roughly and arbitrarily. We set the stroke of the compensation module to be greater than these deviations. Therefore, uncertainties due to calibration deviations were covered and compensated for.

**Figure 7:**
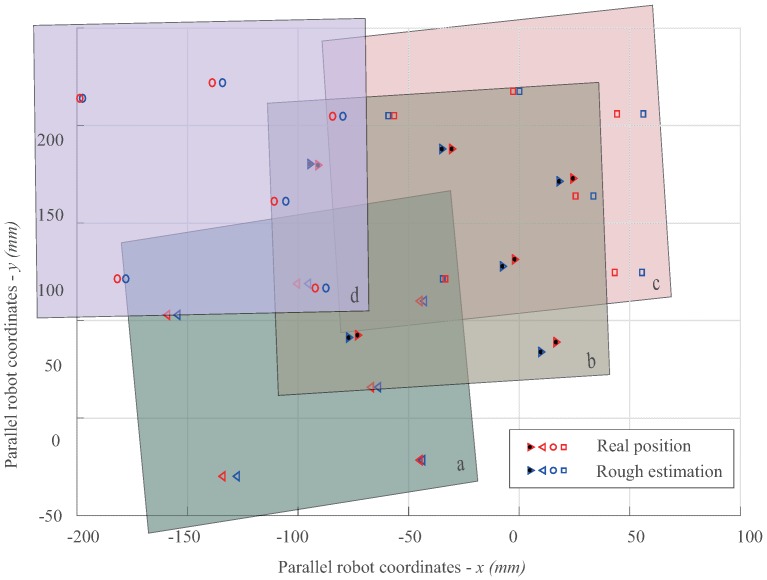
Estimation of holes with respect to different positions of the workpiece under the same rough calibration.

Table 2 shows the result for the second evaluation, which is shown in Figure 7. It is obvious that with the same calibration, the deviation in the estimation error corresponded to the position of the workpiece. This suggests that even if we obtain very good calibration for a given position of the workpiece, there persists the problem of mapping error if the position of the workpiece is changed. Therefore, if we take into account the uncertain placement of the workpiece, mapping through accurate calibration does not necessarily lead to high-precision positioning. To this end, the methodology of this study that we do not seek the way of accurate mapping through costly calibration, but, on the contrary, a coarse-to-fine strategy that only involves rough calibration, is easy to follow.

#### 3.4.2. Result of Dynamic Peg-and-Hole Alignment

**Figure 8:**
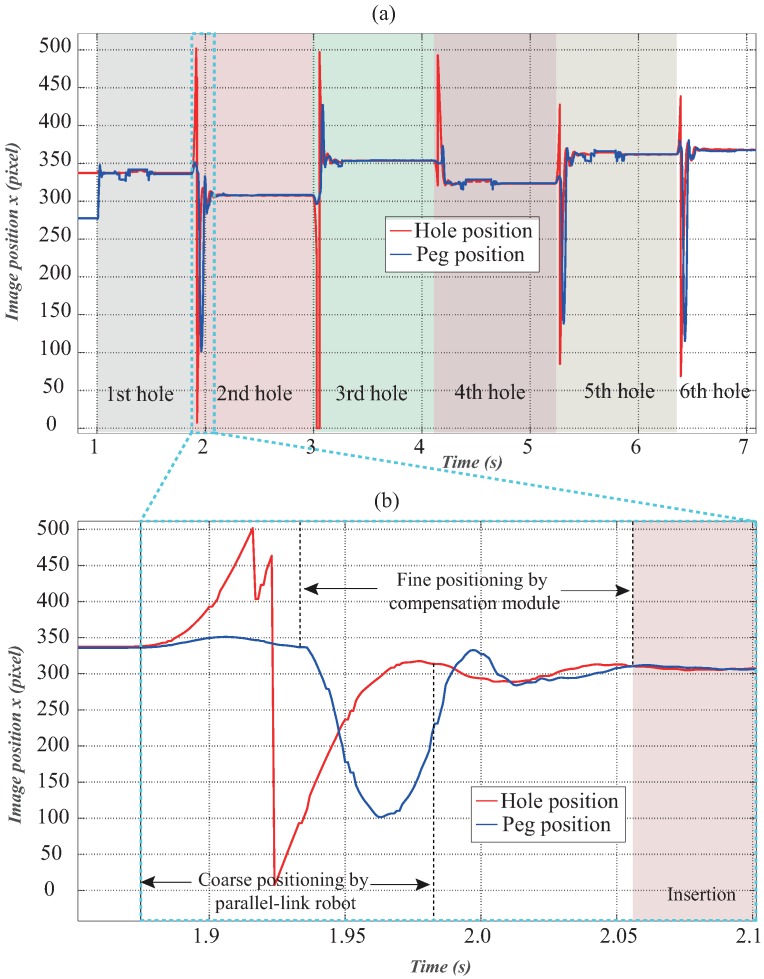
Image feature profiles of peg-and-hole alignment process. (**a**) One experimental trial of continuous peg-and-hole alignment for six holes; (**b**) Zoomed details of the second alignment.

The result of one experimental trial for continuous peg-and-hole alignment for six holes in the workpiece is shown in Figure 8a. The workpiece was placed randomly. Figure 8b shows the details of the second alignment. It can be seen that while the parallel-link robot executed coarse positioning at a high speed (command speed: 2000 mm/s), the hole’s image position from high-speed vision in the *x*-direction did not become stable until after 2.1 s due to the fact that its rotational axis exhibited significant backlash. Nevertheless, the compensation module realized fine alignment within 0.2 s within an accuracy of 0.1 mm. For 20 trials with different positions of the workpiece, all alignments were satisfactory as the proper insertions were observed. The video of several trials of the dynamic peg-and-hole alignment can be found on our website [24].

#### 3.4.3. Discussion

In this dynamic peg-and-hole alignment task, we did not check whether satisfactory alignment can be realized while the workpiece was subject to dynamical disturbances from the environment. The reason for not doing so lay in the difficulty of image recognition for the peg and hole during the insertion period due to occlusions. However, as can be seen in our previous study [18] on the robotic contour tracking task, the same good results can be obtained whether random disturbance by manual vibration is applied on the workpiece or not during task execution. Therefore, we claim that the proposed method is capable of compensating dynamical disturbances from the external environment, as long as its bandwidth is relatively small and within the compensation capability of the add-on module. As for the quantitative analysis on the bandwidth of the add-on module, we would like to summarize it in another article.

## 4. Conclusions

In this paper, we proposed a method to integrate high-speed vision sensing as an add-on module into a general industrial robot to implement fast and accurate position regulation under many uncertainties. Internal uncertainties such as mechanical backlash of the main robot, as well as external uncertainties such as workpiece misalignment or perturbations, are supervised by an eye-in-hand-configured high-speed camera and further compensated for by the add-on module. The main robot’s coarse motion planning is fully automated by using image information from another low-cost VGA camera, and no manual teaching effort is involved. The application scenario of dynamic peg-and-hole alignment showed that an accuracy of 0.1 mm in position regulation was achieved at high stability (100% success rate of insertion) for random placement of the workpiece, which verified the effectiveness of the proposed method. The proposed coarse-to-fine method is compatible with all existing commercial industrial robots due to its independent control scheme. It is easy to implement by simply attaching the add-on module (including high-speed vision and a high-speed compensation actuator with the necessary degrees of freedom) to the main robot’s end effector. The proposed method can be a satisfactory solution in upgrading some low-cost industrial robots with poor accuracy to attain better productivity and task flexibility.

In this study, we limited the implementation of position regulation to be one dimension by using a one-DoF add-on module. An add-on module with three-DoF is being designed, and we intend to test it in the near future. Issues concerning the coupled dynamics of the main robot and the add-on module also need to be further addressed in future work.

## Figures and Tables

**Table 1:** Specifications of the compensation actuator.

Stroke	Maximum Velocity	Maximum Acceleration	Weight
100 mm	1.6 m/s	200 m/s^2^	0.86 kg

**Table 2:** Estimation of holes (*x*-direction, unit: mm) with different positions of the workpiece under the same rough calibration.

Hole Number		Pose a	Pose b	Pose c	Pose d
1	Real	−134.017	−34.608	−181.741	−73.185
	Estimation	−127.544	−33.456	−177.837	−77.154
	**Error**	**6.473**	**1.152**	**3.904**	**−3.969**
2	Real	−44.895	55.573	−92.3	16.625
	Estimation	−43.554	43.335	−87.308	9.838
	**Error**	**1.341**	**−12.238**	**4.992**	**−6.787**
3	Real	−66.912	33.529	−110.702	−2.103
	Estimation	−63.58	25.579	−105.454	−7.514
	**Error**	**3.332**	**−7.95**	**5.248**	**−5.411**
4	Real	−44.886	56.154	−84.365	24.235
	Estimation	−42.865	44.194	−79.821	18.003
	**Error**	**2.021**	**−11.96**	**4.544**	**−6.232**
5	Real	−100.314	0.154	−138.715	−30.644
	Estimation	−95.282	−2.808	−134.139	−35.014
	**Error**	**5.032**	**−2.962**	**4.576**	**−4.37**
6	Real	−158.974	−59.192	−198.655	−90.696
	Estimation	−154.371	−56.455	−197.503	−94.610
	**Error**	**4.603**	**2.737**	**1.152**	**−3.914**
